# Burden of post–acute COVID-19 sequelae in healthcare workers and its course over a 30-month period–results from a prospective multicentre cohort

**DOI:** 10.1007/s15010-024-02418-3

**Published:** 2024-11-12

**Authors:** Tamara Dörr, Carol Strahm, Sabine Güsewell, Tala Ballouz, Emina Kocan, Alexia Cusini, Stephan Goppel, Fabian Grässli, J. Carsten Möller, Milo A. Puhan, Lorenz Risch, Markus Ruetti, Matthias Schlegel, Reto Stocker, Matthias von Kietzell, Danielle Vuichard-Gysin, Stefan P. Kuster, Christian R. Kahlert, Philipp Kohler, for the SURPRISE (SURveillance of infectious diseases among health Professionals In SwitzErland) Study Group

**Affiliations:** 1 Division of Infectious Diseases, Infection Prevention and Travel Medicine, Division of Infectious Diseases and Hospital Epidemiology, Cantonal Hospital St Gallen, Rorschacherstrasse 95, 9007 St. Gallen, Switzerland; 2 Epidemiology, Biostatistics and Prevention Institute, University of Zurich, Zurich, Switzerland; 3 Geriatric Clinic St, Gallen, St. Gallen, Switzerland; 4 Division of Infectious Diseases, Cantonal Hospital Graubünden, Chur, Switzerland; 5 Department of Psychiatry, Clienia Littenheid, Littenheid, Switzerland; 6 Center for Neurological Rehabilitation, Zihlschlacht, Switzerland; 7 Labormedizinisches Zentrum Dr Risch Ostschweiz AG, Buchs, Switzerland; 8 Private Universität Im Fürstentum Liechtenstein, Triesen, Liechtenstein; 9 Centre of Laboratory Medicine, University Institute of Clinical Chemistry, University of Bern, Inselspital, Bern, Switzerland; 10 Fuerstenland Toggenburg Hospital Group, Wil, Switzerland; 11 Hirslanden Clinic, Zurich, Switzerland; 12 Hirslanden Clinic Stephanshorn, St. Gallen, Switzerland; 13 Division of Infectious Diseases and Hospital Epidemiology, Thurgau Hospital Group, Muensterlingen, Switzerland; 14 Department of Research and Development, Swiss National Centre for Infection Prevention (Swissnoso), Berne, Switzerland; 15 Department of Infectious Diseases and Hospital Epidemiology, Children’s Hospital of Eastern Switzerland, St. Gallen, Switzerland

**Keywords:** SARS-CoV-2, Post-acute sequelae of COVID-19, Long COVID, Healthcare workers, Disease burden

## Abstract

**Purpose:**

As healthcare workers (HCW) have been disproportionally affected by COVID-19, its post-acute sequelae (PASC) in HCW can impact healthcare systems. We assessed the burden and course of PASC in HCW over a 30-month period.

**Methods:**

In a prospective multicentre HCW cohort in Switzerland, PASC surveys were conducted in 03/2021, 09/2021, 06/2022, 04/2023, and 10/2023. Stratified by viral variant at first infection, the prevalence of PASC symptoms, self-experienced PASC and the Post-COVID Functional Status (PCFS) were analysed cross-sectionally in 10/2023, self-perceived success of therapeutic measures used was assessed. The evolution of PASC symptoms and PCFS in Wild-type and non-Wild-type infected HCW compared to uninfected controls was analysed longitudinally across all surveys.

**Results:**

In cross-sectional analysis, 1704 HCW (median age 47 years, 82.2% female) were included. Thereof, 30.7% reported ≥ 1 PASC symptom in 10/2023, with 115 (6.7%) stating to have or have had PASC. Both were most common after Wild-type infection compared to other variants. Overall, 17/115 (15%) indicated relevant/severe restrictions in their daily activities and of 85 (74%) that tried ≥ 1 measure against their symptoms, 69 (81%) reported having benefitted. Longitudinal analysis (n = 653) showed a significantly higher proportion of Wild-type infected HCW to report PASC symptoms compared to controls in 03/2021 (+ 21%, 95% CI 4–39), with decreasing trend (+ 7%, 95%CI -10–25 in 10/2023). This effect was not evident for non-Wild-type infected HCW.

**Conclusions:**

Over a 30 month period, overall PASC burden in our HCW cohort decreased, although 1% still experience relevant restrictions in their daily life; Wild-type infected individuals show the highest disease burden.

**Supplementary Information:**

The online version contains supplementary material available at 10.1007/s15010-024-02418-3.

## Introduction

Post-acute sequelae of COVID-19 (PASC) have emerged as serious health problem during the SARS-CoV-2 pandemic carrying the potential for temporal or long-term disability [1]. As healthcare workers (HCW) have been disproportionally affected by COVID-19 and the burden of PASC has been suggested to be particularly high in this population [2], the fear of work force losses in the healthcare system has been raised [3]. Given that prolonged symptoms after SARS-CoV-2 infection occur in 4 to 45% [4, 5] of individuals with in some cases significant impact on their work ability [6–8], this poses a considerable threat on societies worldwide. While knowledge on the impact of PASC and its natural course is increasing [9–11], longitudinal studies assessing long-term outcomes and symptom evolution, especially beyond the initial post-infection months, remain scarce.

Given the critical role of HCW and the potential implications of PASC on their health, this study aims to assess the PASC burden in a HCW population as of October 2023, to describe the self-perceived benefit of different therapeutic measures used against PASC, and to assess the temporal evolution of PASC symptoms over 30 months.

## Methods

### Setting and participants

This study is nested in a previously described multicentre open cohort that prospectively follows HCW (defined as any hospital employee with or without patient contact ≥ 16 years) from healthcare institutions in North-Eastern Switzerland since 08/2020 [12]. All participants provided electronic consent. The study was approved by the Ethics Committee of Eastern Switzerland (#2020–00502).

### Baseline and PASC questionnaires

**Fig. 1:**
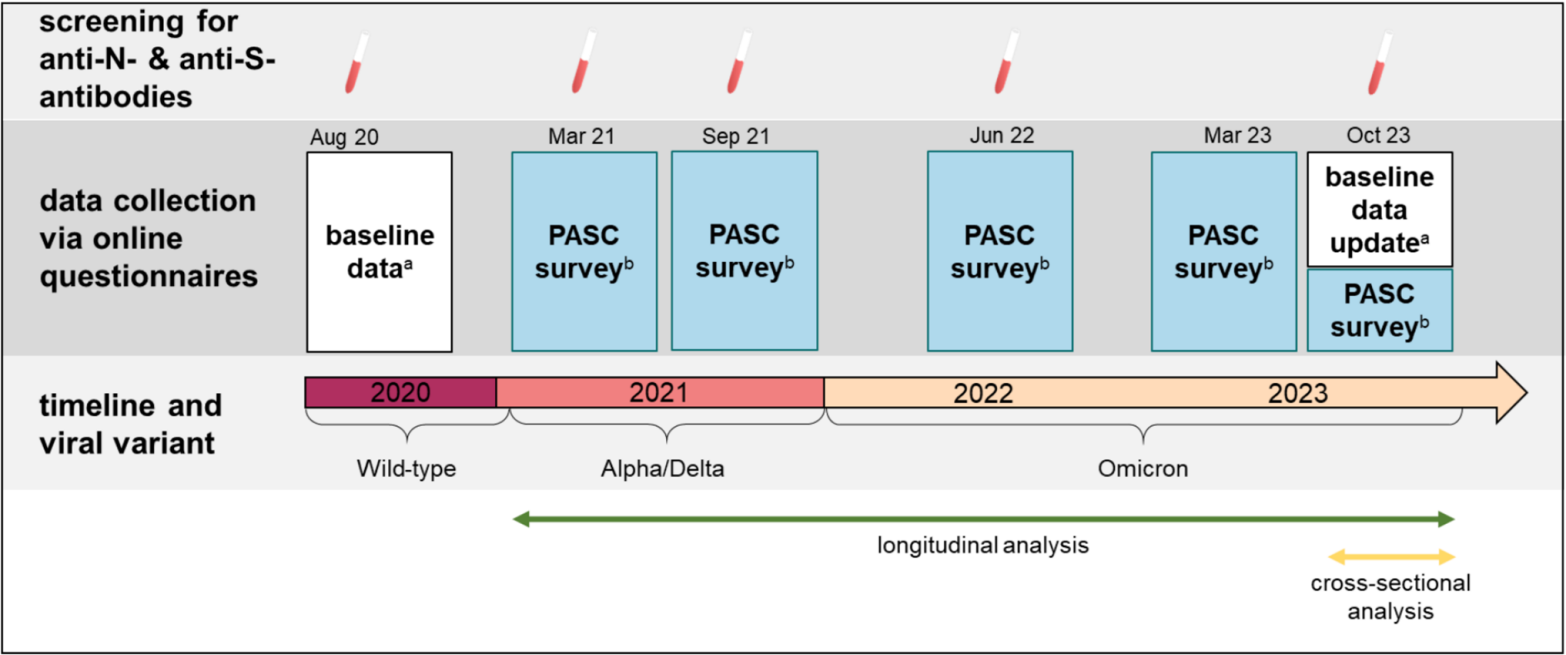
Overview study timeline and elements included in the analyses. ^a^data on demographics, health and social determinants. ^b^presence of PASC symptoms, Fatigue Severity Scale, SARS-CoV-2 vaccination and infection history

At inclusion, baseline data (i.e. age, gender, health determinants, occupational factors and social life parameters) were obtained through an online questionnaire and updated in October 2023. Five PASC surveys were conducted in March 2021, September 2021, June 2022, March 2023 and October 2023 (Fig. 1). At each, participants updated their history on SARS-CoV-2 vaccination (date/type of any vaccine received) and infection (date of any positive swab and > 30 days after a previous positive test) and answered questions on PASC-related parameters. To avoid contamination of self-reported PASC with acute symptoms, questionnaires completed within 28 days of a positive SARS-CoV-2 test or within 7 days of SARS-CoV-2 vaccination were excluded (Figure S1).

All surveys included the presence of selected PASC-compatible symptoms and the 9-item Fatigue Severity Scale (FSS) [13]. Since September 2021, we added questions on presence of current self-perceived PASC and on its impact on daily life indicated on the 5-point Post-COVID-19 Functional Status Scale (PCFS) [14]; since March 2023, we also asked about improvement or full recovery from previous PASC. In October 2023, participants were asked which therapeutic measures they had tried and if they found them to be beneficial (Table S1) [15]. Along with the PASC surveys (except March 2023), participants provided serum samples and were screened for anti-nucleocapsid (anti-N) antibodies [16].

### Main predictor and outcomes

We evaluated (i) cross-sectional data from participants answering the PASC survey in October 2023 (referred to as cross-sectional analysis) and (ii) longitudinal data from the subset of HCW who completed all five PASC surveys (referred to as longitudinal analysis) (Fig. 1). Because of its pivotal role on the development of PASC, HCW were stratified by the SARS-CoV-2 variant most likely to have caused the first infection [16, 17] to “Wild-type” (between 01/2020 and 01/2021), “Alpha/Delta” (between 02/2021 and 12/2021, combined due to small numbers), “Omicron” (between 01/2022 and 09/2023) or uninfected (i.e. no positive swab, anti-N-negative). HCW not attributable to a viral variant (i.e. anti-N positivity at inclusion without reporting a positive test) were excluded and re-infections were ignored, because we found no impact on PASC in a previous analysis [18]. Methods for attribution and validation of stratifications are detailed in the Supplement.

For the cross-sectional analysis, the main outcomes were the proportions of HCWs who (i) indicated ≥ 1 persisting PASC-related symptom, (ii) self-reportedly ever experienced PASC (current PASC and fully resolved PASC) and their (iii) functional restrictions in daily life due to PASC according to the PCFS. We also evaluated the self-perceived evolution of PASC symptoms (i.e. resolved, partially resolved, unchanged/worsened), and the perceived success of any therapeutic measures tried against PASC. For the longitudinal analysis, we assessed the time course of PASC symptom prevalence, the FSS, and the PCFS across questionnaires.

### Statistical analyses

We used descriptive statistics to characterize participants in the cross-sectional and the longitudinal analysis, respectively and compare those in the cross-sectional analysis with participants that dropped out between June 2022 and October 2023. In the cross-sectional analysis, binary outcomes were analysed using univariable logistic regression with viral variant as predictor. Models without intercept were fitted to estimate symptom prevalence with 95% likelihood ratio confidence intervals for each group. Models with intercept were run to obtain odds ratios (OR) with 95% likelihood ratio confidence intervals (CI) for the comparison of each viral variant with the uninfected control group. PCFS levels were only analysed descriptively by calculating the relative frequency of each category.

To assess the consistency and plausibility of self-reported outcomes, we compared the number of PASC symptoms among HCW with different self-reported PASC experience (none, resolved or current PASC) and with different function limitations (PCFS levels) using the cross-sectional data and negative binomial models.

In the longitudinal analysis, logistic regression models for symptom prevalence and negative binomial models for mean FSS, respectively, were fitted separately for each time point, and trends across time points were evaluated qualitatively. Differences between Wild-type infected participants and uninfected controls are reported relative to the uninfected group along with 95% CI which were obtained from standard two-sample proportion tests and t-tests. Non-Wild-type infected participants were pooled into one group, for which no statistics are reported, because these infections occurred during the longitudinal follow-up. PCFS levels were analysed descriptively by calculating the relative frequency of each category at each time point.

All analyses were performed using R statistical software Version 4.2.2. and the STROBE reporting guidelines for observational studies were adhered to [19].

## Results

### Study population and drop-outs

Overall, 1935 HCW answered the questionnaire in October 2023, with 74 (3.8%) excluded due to recent infection or vaccination and 261 (13.5%) that were not attributable to a viral variant, resulting in 1704 (88.1%) HCW which were included in the cross-sectional analysis. Of these, 653 (38.3%) had answered all five PASC surveys and were included in the longitudinal analysis. Baseline characteristics of cross-sectional and longitudinal populations are summarized in Table 1.

**Table 1:** Baseline characteristics of participants included in the cross-sectional analysis and in the longitudinal analysis

	**HCW in cross-sectional analysis**		***missing***	**Subgroup of HCW in longitudinal analysis**		***missing***
n	1704			653		
Personal characteristics						
Age (in years), median (IQR)	47	(38–55)	0	48	(40–56)	0
Sex, female (%)	1400	(82.2)	0	534	(81.8)	0
Ethnicity, caucasian (%)	1683	(98.9)	0	647	(99.1)	0
Body mass index, median (IQR^a^**)**	23.6	(21.5–26.8)	1	23.7	(21.5–26.6)	1
≥ 1 Comorbidity (%)	811	(47.6)	0	337	(51.6)	0
≥ 1 Medication (%)	552	(32.4)	0	227	(34.8)	0
Active smoker (%)	246	(14.4)	0	98	(15.0)	0
Alcohol > 1 drink/week (%)	645	(39.8)	84	269	(42.8)	25
Occupational characteristics						
Profession			0			0
Physician (%)	222	(13.0)		95	(14.5)	
Nurse (%)	849	(49.8)		332	(50.8)	
Other (%)	633	(37.1)		226	(34.6)	
Patient contact in last 4 weeks (%)	1200	(70.4)	0	485	(74.3)	0
> 0.8 FTE^b^ (%)	775	(45.5)	0	292	(44.7)	0
SARS-CoV-2^c^ vaccination			0			0
None (%)	172	(10.1)		71	(10.9)	
1–2 doses (%)	382	(22.4)		123	(18.8)	
≥ 3 doses (%)	1150	(67.5)		459	(70.3)	
SARS-CoV-2^c^ infections			0			0
None (%)	122	(7.2)		50	(7.7)	
Wild-type (%)	271	(15.9)		112	(17.2)	
Alpha/Delta (%)	213	(12.5)		68	(10.4)	
Omicron (%)	1098	(64.5)		426	(64.7)	
Anti-N^d^-positivity October 2023 (%)	1219	(89.9)	348	491	(90.1)	108

^a^IQR = interquartile range

^b^FTE = full-time equivalent

^c^SARS-CoV-2 = severe acute respiratory syndrome coronavirus type 2

^d^Anti-N = anti-nucleosid-antibodies

Development of the cohort since its inception in August 2020 is summarized in Figure S1. Of 3522 HCW included in June 2022, 1587 HCW (45.1%) were considered drop-outs. Comparing these to the cross-sectional cohort (n = 1704) in terms of PASC symptoms and FSS in June 2022, drop-outs had more PASC symptoms (overall RR 1.22, 95% CI 1.10–1.36) and higher FSS (overall RR 1.04, 95% CI 1.00–1.08) (Figure S2).

### Prevalence of PASC and reported severity in October 2023

In the 1704 included individuals, median age was 47 years (interquartile range, IQR 38–55) and most (82.2%) were female; 1432 (84.0%) reported ≥ 1 SARS-CoV-2 infection (median time since infection 20.2 months); anti-N seroconversion between 2022 and 2023 was documented for an additional 150 (8.8%) individuals; 122 (7.2%) participants were classified as uninfected controls.

**Fig. 2:**
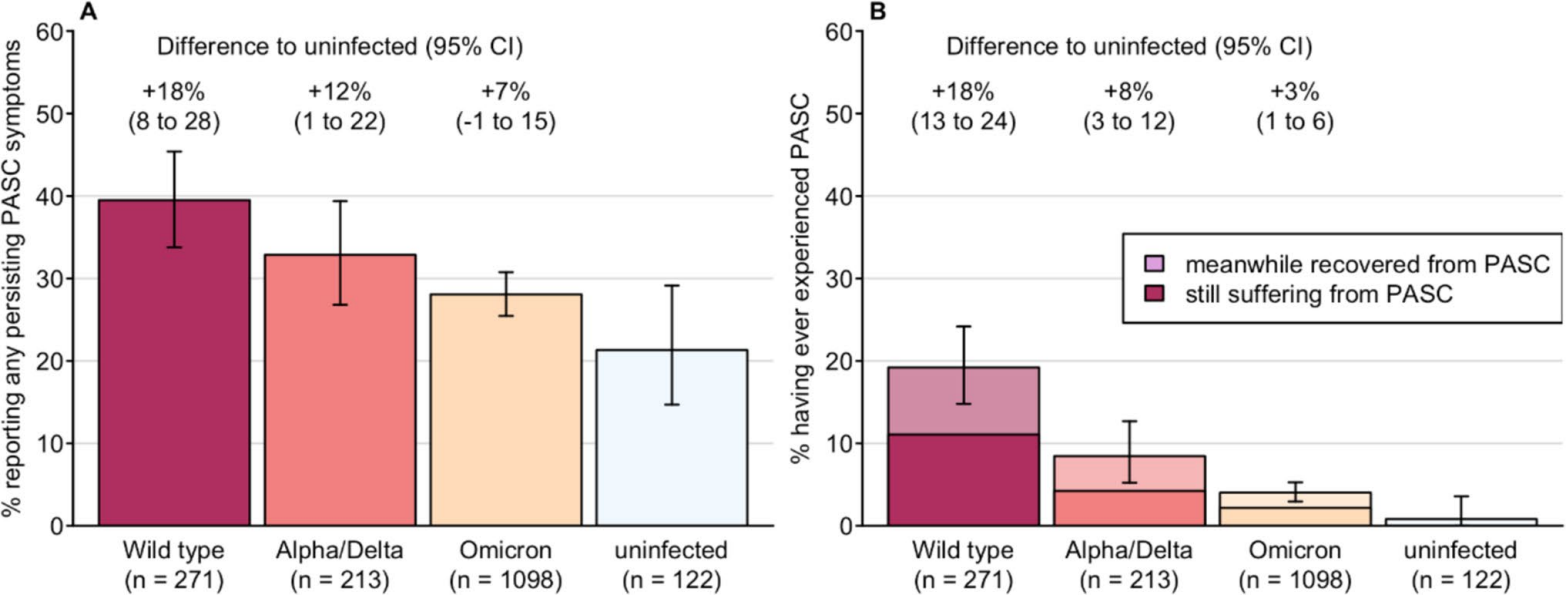
**A** Prevalence of PASC symptoms in healthcare workers in October 2023 and **B** self-reported PASC experience (current or resolved PASC) by dominating viral variant at first infection. Percentage difference in relation to the uninfected control group and 95% CI were derived from standard two-sample proportion tests and t-tests

While 551/1704 (30.7%) of HCW reported ≥ 1 PASC symptom, only 115 (6.7%) indicated to currently have or to previously have had PASC. These figures were most common after Wild-type (39% and 19.2% respectively), followed by Alpha/Delta (33% and 8.5%) and Omicron infection (28% and 4.0%), and were significantly more common compared to uninfected controls (21% and 0.8%) (Fig. 2). Of the 115 participants with self-reported PASC experience, 63 (55%) indicated to still suffer from PASC (Fig. 2B), and 17 (14.8%) indicated relevant or severe restrictions in their daily activities due to PASC, corresponding to 1.0% of the total (n = 1704) population. These proportions showed no remarkable differences among viral variants (Fig. 2). Self-reported PASC experience and restrictions in daily activities correlated positively with the number of PASC symptoms reported in October 2023 (Figure S3).

### Evolution of PASC and therapeutic measures taken in October 2023

A majority of 74.0% (85/115) indicated having taken ≥ 1measure against their symptoms and 69 of these (81%) reported having benefitted from at least one measure. Psychotherapy, ergotherapy and smoking cessation were reported to reduce PASC symptoms by all who tried those measures, followed by complementary medicine (17/21, 81% reporting symptom reduction), the use of antihistamines (4/5, 80%) and pacing (12/17, 71%). For yoga (6/16, 38%) or SARS-CoV-2 vaccination (2/13, 15%), these figures were lower (Figure S3).

### Longitudinal analysis of PASC evolution

Of the 653 HCW included, 112 (17.2%) were attributed to Wild-type and 491 (75.2%) to non-Wild-type infection; 50 (7.7%) were considered uninfected.

**Fig. 3:**
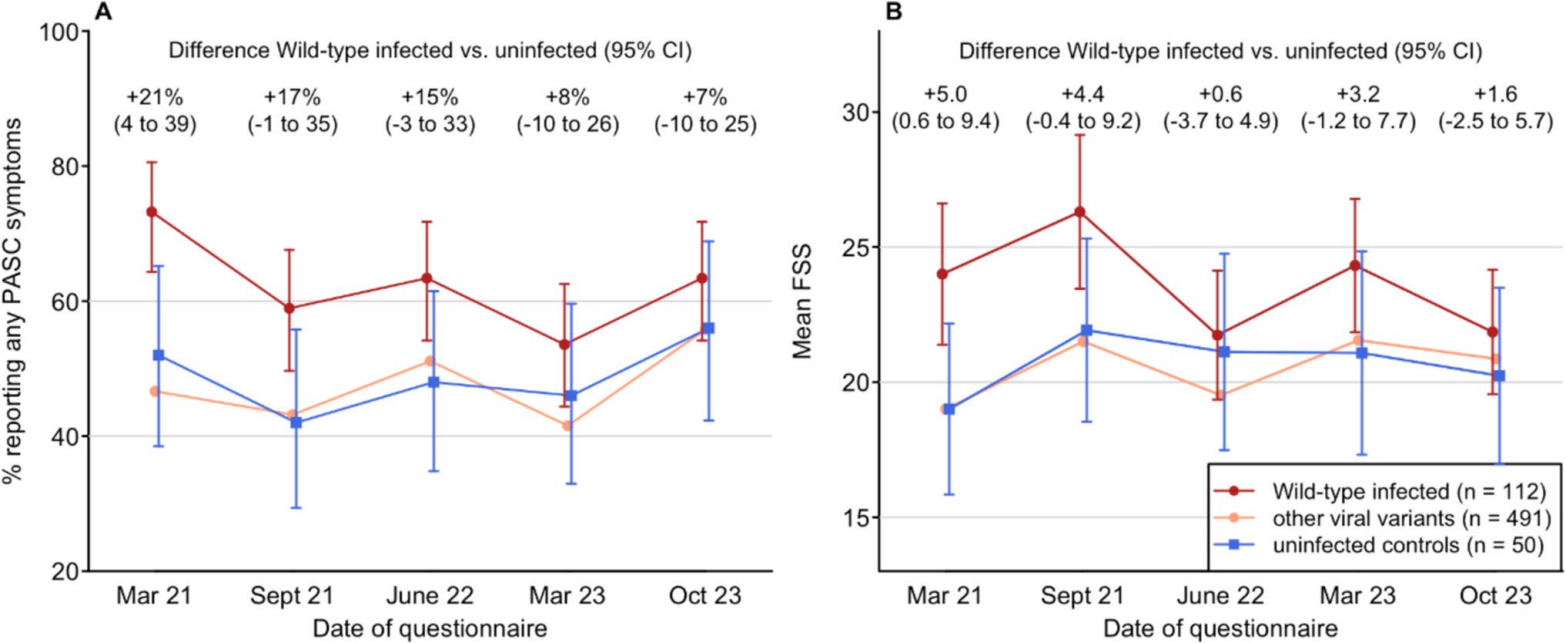
Longitudinal analysis showing **A** proportion of healthcare workers with any PASC symptoms, and **B** mean fatigue severity score by viral variant across the five questionnaires. Unadjusted ratios between Wild-type infected and uninfected participants with 95% CI were were derived from standard two-sample proportion tests and t-tests. Other viral variants: Non-Wild-type viral variants were pooled and are shown without statistics for the sake of clarity

The proportion of participants reporting any PASC symptoms was consistently larger in Wild-type infected individuals than in non-Wild-type infections and uninfected controls, with a difference in proportions to of + 21% 95% CI (4–39%) in March 2021, decreasing to + 7% (95% CI -10–25%) in October 2023 (Fig. 3A). Likewise, the difference of mean FSS decreased from + 5% (95% CI 0.6–9.4%) to + 1.6% (95% CI -2.5–5.7%) for Wild-type infected compared to uninfected controls (Fig. 3B). For non-Wild-type infections, this effect could not be demonstrated.

The proportion of participants reporting to suffer from PASC slightly decreased over time, from 5.2% in September 2021 to 4.4% in October 2023. This development was most pronounced in the Wild-type infected individuals with a decrease from 22.6 to 13.9%.

**Fig. 4:**
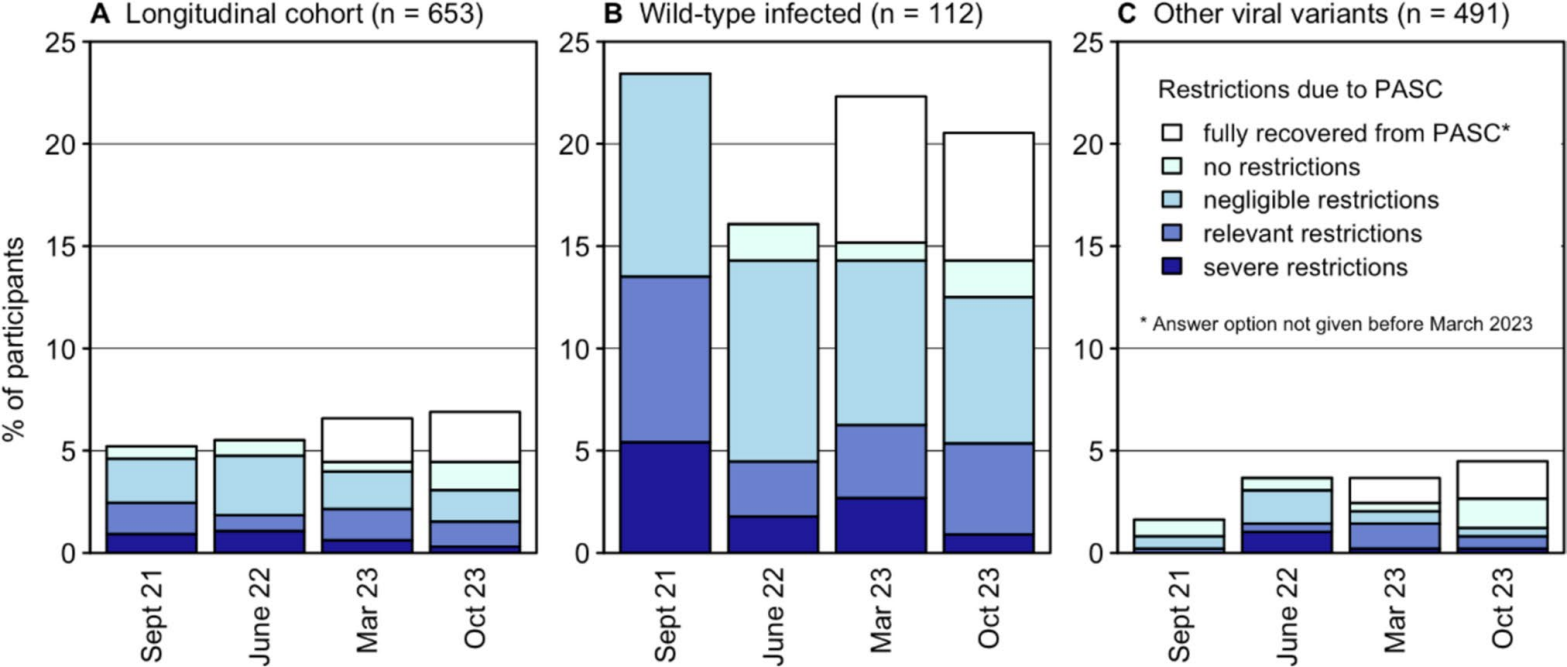
Evolution of Post Covid Functional Scale (PCFS) from September 2021 to October 2023 **A** in the longitudinal cohort (n = 653), **B** in Wild-type infected participants of the longitudinal cohort, and **C** in participants first infected by other viral variants. Participants were asked whether they currently suffer from PASC, and if so, how much their daily life was affected (PCFS)

The attributed restrictions in daily life activities measured with PCFS likewise decreased continuously, with 2.5% of all participants indicating relevant restrictions in September 2021 and 1.5% in October 2023 (Fig. 4A), the figures being 13.0% and 5.2% for the Wild-type infected, respectively (Fig. 4B).

## Discussion

### Key findings

Among our HCW, around 1% of over 1700 individuals reported relevant limitations of daily activities due to PASC after a median time since first infection of 20.2 months. While PASC burden is highest after Wild-type infection, the proportion of individuals with relevant PASC-compatible symptoms has been decreasing over the observation period of 30 months and is similar to uninfected individuals in October 2023. A majority of affected HCW reported the use of non-evidence based measures to reduce the symptoms of PASC, with variable self-reported benefits.

### Prevalence, role of viral variant, disease burden

Over 30% of HCW reported persisting PASC-compatible symptoms since their SARS-CoV-2 infection after a median follow-up of 20 months, which is in line with other cohort studies [2, 20–22]. Due to the non-specific nature of many symptoms and the use of different diagnostic criteria, others suggested the prevalence might be overestimated if based on symptoms alone [9]. This is reflected in our data with only 6.7% of participants stating to currently or previously suffering from PASC. Nonetheless, the data available for HCW is in contrast to the prevalence of persistent symptoms reported for the general population, that is estimated to be 15% at 12 months after infection [4, 7–9, 11]. While the probably greater health literacy and awareness in a HCW cohort might contribute to this difference, the fact that HCW were disproportionally affected during the first waves of COVID-19 [2]—and PASC being most common and most pronounced after Wild-type infection—[12, 17], suggests that HCW are indeed more frequently affected by PASC. This poses a considerable threat on healthcare systems where HCW work forces as a resource are of societal relevance. Thus, the 1% that indicated relevant impairment in their daily life due to PASC, which might be even more relevant than the mere presence of symptoms from a healthcare system perspective, can indeed be impactful.

### Evolution over time: self-reported and longitudinal data

Due to the emerging character of the condition, there is to date only limited knowledge about the natural course of PASC. However, most studies could show that a substantial proportion of PASC symptoms alleviate over time [8, 9, 11, 23]. Also in our cohort, a majority of those who reported ever having PASC reported partial or full recovery from their symptoms since onset. While most observations show the major share of improvement to happen in the first 6 months [1, 23] and the majority of symptoms to resolve after 12 months [2, 21, 23, 24], the prognosis beyond 12 months is still unclear. In some large cohorts no relevant improvement beyond 6 and up to 18 months could be seen [9, 23], others observed an ongoing decrease of PASC symptoms beyond 15 and up to 22 months [25]. We also observed the most pronounced drop in ratios compared to uninfected controls in the first 6 months after infection, but further decrease was seen up to 30 months. Although follow-up time varied, we have shown earlier that the prevalence of PASC in our cohort is mainly driven by Wild-type infected individuals with consistent follow-up time, indicating that further improvement of PASC can be expected beyond 12 months. Importantly, the proportion of individuals with relevant functional impairment due to PASC has also been decreasing over time.

### Potential beneficial effects of measures used by participants to alleviate PASC symptoms

We found that the majority of those self-reportedly suffering from PASC tried at least one potential therapeutic measure and most reported to have benefited. While scientific evidence to date is sparse for most strategies, current guidelines include titrated increase of physical activities and psychological strains [15, 26]. Corresponding measures such as pacing, stress reduction and increased phases of rest as well as physiotherapy, were tried by the majority of our PASC cohort but showed variable efficacy of about 60–70%. Interestingly, a high proportion of those who tried complementary medicine as well as antihistamines attributed improvement to these measures, scientific evidence on pharmacologic or nutritional strategies to date is however sparse and contradictory [26, 27]. Similarly, some have reported beneficial effects for SARS-CoV-2 vaccination [28, 29], but others have found little to none, comparable to our results [30]. High-quality interventional studies are urgently needed to explore these effects.

## Strengths and limitations

Important strengths of our study are the clearly defined infection and vaccination status for each individual, the multi-dimensional exploration of PASC, and the availability of longitudinal data over 30 months for a subgroup of our participants. Some limitations should however be considered. First, the high number of drop-outs which had more PASC symptoms and higher FSS, likely leads to underestimation of PASC prevalence and burden. Second, the inclusion of only HCW might limit applicability of our findings to other populations. Also, the possibility of false negative or false positive results in both, swab tests and serology assays would have led to misclassification and influence the difference estimates between the groups. Last, since the definition of PASC has changed over time and is still not used consistently by the scientific community, comparability to other studies is limited.

## Conclusion

Our data show a steady and continuing decrease of PASC prevalence and burden in an HCW population of predominantly female, and previously healthy individuals over the course of 30 months. Choice and benefit of therapeutic measures vary considerably, however, recovery rate is high after 30 months and seems to be time-dependent.

## Supplementary Information

Below is the link to the electronic supplementary material.

Supplementary file1 (DOCX 396 KB)

## Data Availability

No datasets were generated or analysed during the current study.
