# Supplementary material for: Burden of post–acute COVID-19 sequelae in healthcare workers and its course over a 30-month period–results from a prospective multicentre cohort
Source: Infection. 2024 Nov 12;53(4):1311–9. doi: 10.1007/s15010-024-02418-3 (PMC12316709; doi:10.1007/s15010-024-02418-3)
Supplement: Supplementary file 1 — Supplementary file1 (DOCX 396 KB) [file 15010_2024_2418_MOESM1_ESM.docx]

**SUPPLEMENTS**

**Case definition of viral variant attribution**

Participants were stratified to the viral variant according to the locally predominant strain at the date of a participant's first positive swab test. Detection of SARS-CoV-2 was made by either polymerase chain reaction or rapid antigen test, depending on the method used in the participating institution or, in case of home testing, by the participant. Locally predominance was inferred from sequencing data from Northeastern Switzerland as available from the Federal Office of Public Health of Switzerland.

Because SARS-CoV-2 testing was mostly abandoned after June 2022, we considered anti-N seroconversions occurring between 07/2022 and 09/2023 as indicative of Omicron infection even if no positive test was reported by the participant.

HCW that never reported a positive swab for SARS-CoV-2 and whose serum samples were repetitively negative for anti-N were considered as uninfected.

**Validation of self-reported tests**

Self-reported swab test results were validated for a subset between June and September 2020 when testing and reporting to the local occupational health division was obligatory. All self-reported positive tests and a random selection of self-reported negative tests during this time period were cross-checked with the respective database of the occupational health division in the largest participating institution.

**Supplementary Tables**

**Table S1**. Elements of the Post-acute sequelae of SARS-CoV-2 (PASC) questionnaires at individual timepoints.

|  | **Mar 21** | **Sep 21** | **Jun 22** | **Mar 23** | **Oct 23** |
| --- | --- | --- | --- | --- | --- |
| **PASC symptoms asked** |  |  |  |  |  |
| Dyspnoea | ✓ | ✓ | ✓ | ✓ | ✓ |
| Cough | ✓ | ✓ | ✓ | ✓ | ✓ |
| Headache | ✓ | ✓ | ✓ | ✓ | ✓ |
| Feverish feeling / fever | ✓ | ✓ | ✓ | ✓ | ✓ |
| Chills | ✓ | ✓ | ✓ | ✓ | ✓ |
| Limb/muscle pain | ✓ | ✓ | ✓ | ✓ | ✓ |
| Anorexia | ✓ | ✓ | ✓ | ✓ | ✓ |
| Hair loss | ✓ | ✓ | ✓ | ✓ | ✓ |
| Joint pain | ✓ | ✓ | ✓ | ✓ | ✓ |
| Chest pain | ✓ | ✓ | ✓ | ✓ | ✓ |
| Increased palpitations | ✓ | ✓ | – ^a^ | ✓ | ✓ |
| Impaired taste or olfaction | ✓ | ✓ | ✓ | ✓ | ✓ |
| Weakness / tiredness | ✓ | ✓ | ✓ | ✓ | ✓ |
| Dizziness | ✓ | ✓ | ✓ | ✓ | ✓ |
| Exhaustion / burnout | ✓ | ✓ | ✓ | ✓ | ✓ |
| Diarrhea | – | – | ✓ | ✓ | ✓ |
| Stomach pain | – | – | ✓ | ✓ | ✓ |
| Exanthema | – | – | ✓ | ✓ | ✓ |
| Brain fog | – | – | ✓ | ✓ | ✓ |
| Distinction of chronic symptoms that appeared after March 2020 from acute or pre-existing symptoms | no | no | ✓ | ✓ | ✓ |
| **Presence of PASC** |  |  |  |  |  |
| current | – | ✓ | ✓ | ✓ | ✓ |
| experienced but resolved ^b^ | – | – | – | ✓ | ✓ |
| ***Recorded Scores*** |  |  |  |  |  |
| **Fatigue Severity Scale (FSS)** | ✓ | ✓ | ✓ | ✓ | ✓ |
| **Post COVID Functional Scale (PCFS)** | – | ✓ | ✓ | ✓ | ✓ |
| **Other elements** |  |  |  |  |  |
| **Therapeutic measures** |  |  |  |  |  |
| None | – | – | – | – | ✓ |
| Increase of sleep | – | – | – | – | ✓ |
| Rest and reduction of stress | – | – | – | – | ✓ |
| Sports (if yes, which?) ^c^ | – | – | – | – | ✓ |
| Yoga | – | – | – | – | ✓ |
| SARS-CoV-2 vaccination | – | – | – | – | ✓ |
| Physiotherapy | – | – | – | – | ✓ |
| Pacing (energy management) | – | – | – | – | ✓ |
| Antihistamines (i.e. levocetirizine) | – | – | – | – | ✓ |
| Other medication (if yes, which?) ^c^ | – | – | – | – | ✓ |
| Oxygen therapy | – | – | – | – | ✓ |
| Dietary intervention (if yes, which?) ^c^ | – | – | – | – | ✓ |
| Alternative or complementary medicine (if yes, which?) ^c^ | – | – | – | – | ✓ |
| Other (if yes, which?) ^c^ | – | – | – | – | ✓ |

^a^ the symptom “presence of palpitation” was erroneously not asked; the PASC score based on the other 14 symptoms was therefore multiplied with 15/14 and rounded.

^b^ included as additional level in analysis of PCFS

^c^ optional free text form

**Figure S1.** Detailed flow sheet of participants from initiation of the cohort in August 2023 until October 2023.

**
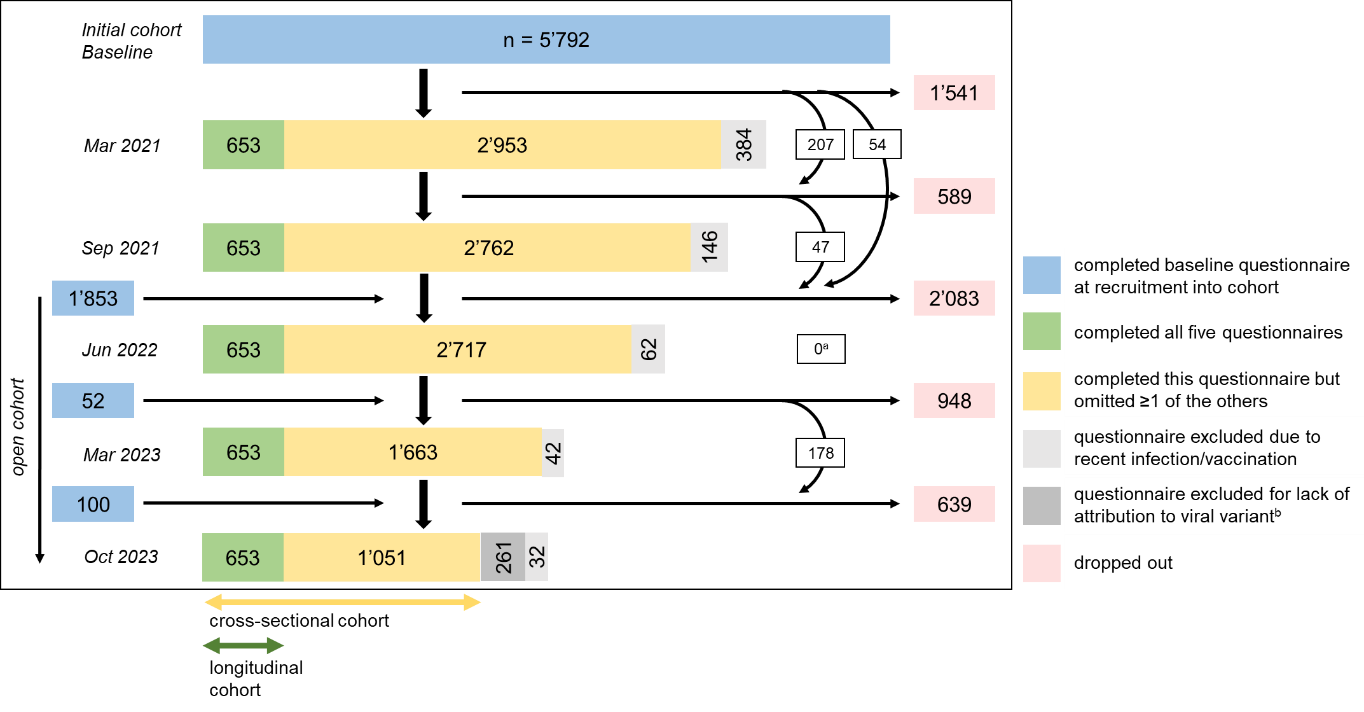
**

^a^ PASC questions were mandatory part of the SURPRISE+ baseline questionnaire.

Participants recruited later appear as new entries at the next time point.

^b^ Exclusion criterion only applied for analysis in Oct 2023.

**Figure S2.** Mean number of PASC symptoms (left) and mean fatigue severity score (right) reported in June 2022 by participants who continued to participate until October 2023 (blue) and those who subsequently dropped out (beige), stratified by viral variant dominating at the time of the first positive swab. Rate ratios (RR) for dropouts vs. continuing participants were determined with negative binomial models across the four groups of viral variants after checking that RRs did not differ significantly between viral variants; 95% likelihood ratio confidence intervals and p-values from Wald tests are reported.

**
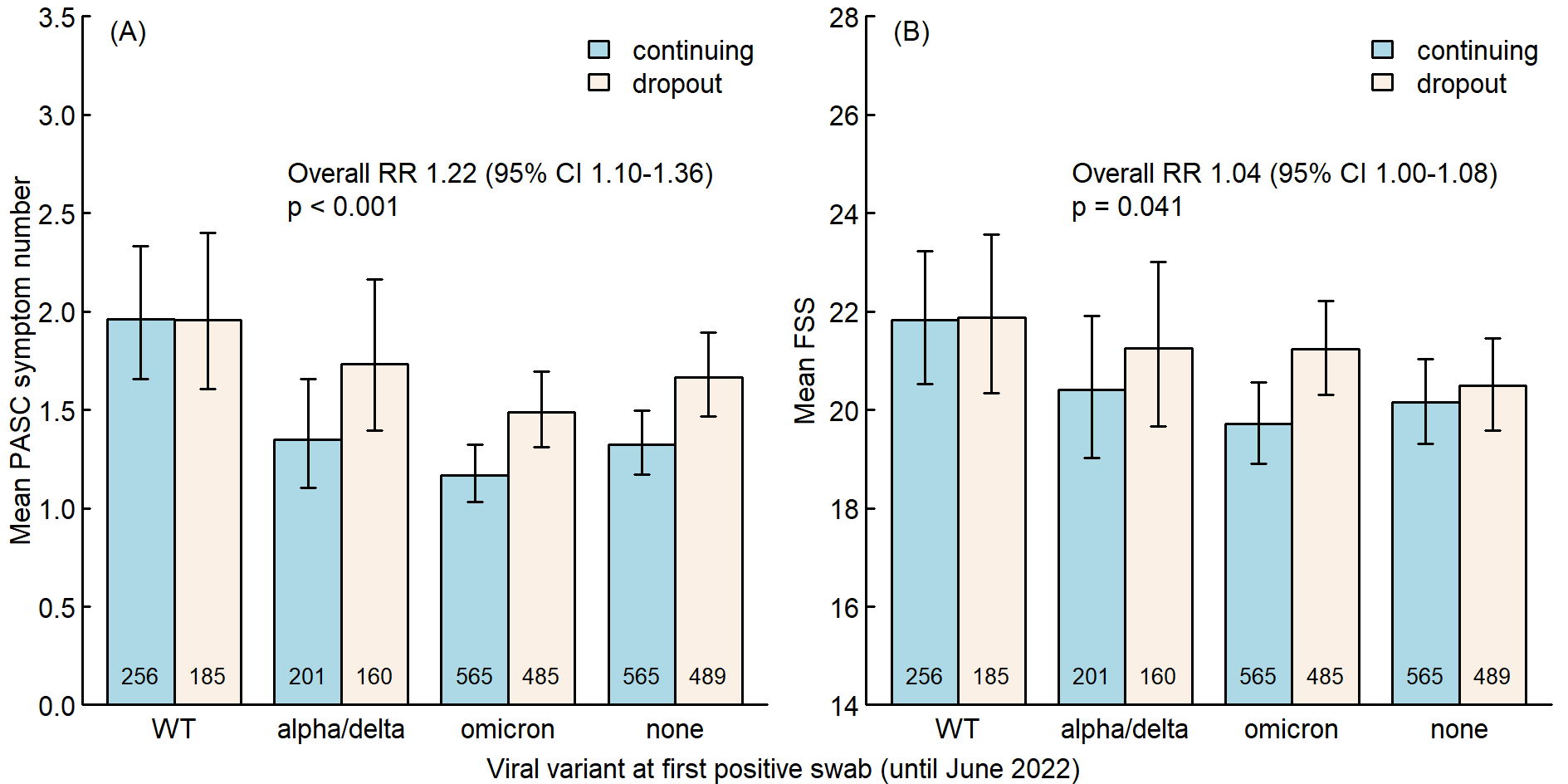
**

**Figure S3.** Mean number of PASC symptoms reported in October 2023 according to (A) PASC experience and (B) restrictions in daily life due to current PASC (n = 63). Numbers above bars indicate the relative increase in mean symptom number relative to the first group ("none"), estimated along with 95% confidence intervals with negative binomial models.
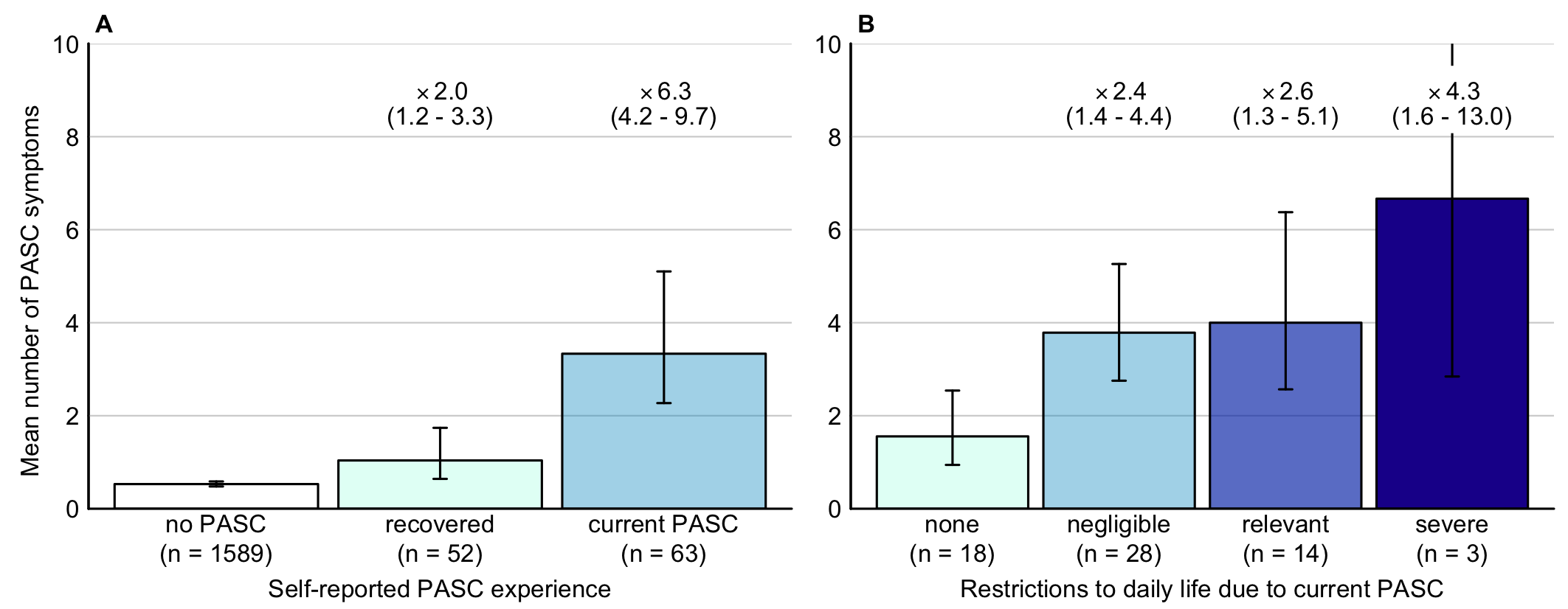


**Figure S4.** Therapeutic measures tried with n = number of HCW who tried them, and proportion of these who achieved an improvement of PASC symptoms through this measure.

**
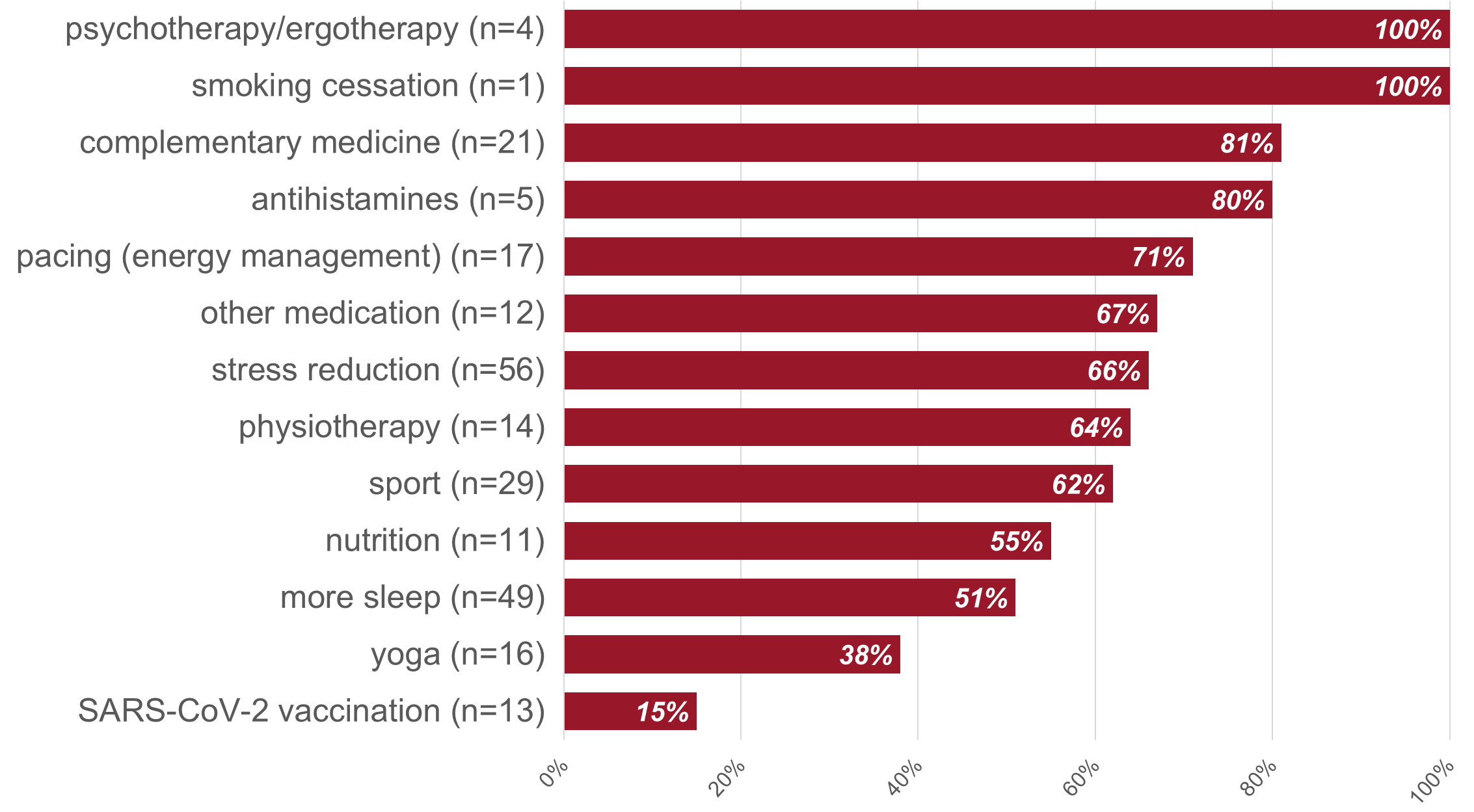
**
